# User Experiences of Limb-Worn Wearable Devices for Monitoring Parkinson Disease Motor Function and Blood Pressure: Usability Study

**DOI:** 10.2196/73423

**Published:** 2025-11-19

**Authors:** Lorna Kenny, Marco Sica, Colum Crowe, Lauren O'Mahony, Brendan O'Flynn, David Scott Mueller, Salvatore Tedesco, John Barton, Suzanne Timmons

**Affiliations:** 1 Centre for Gerontology and Rehabilitation School of Medicine University College Cork Cork Ireland; 2 Tyndall National Institute University College Cork Cork Ireland; 3 AbbVie (United States) North Chicago, IL United States

**Keywords:** Parkinson disease, wearable device, technology, experience, stigma

## Abstract

**Background:**

Wearable devices have the potential to provide reliable and objective assessment and monitoring of Parkinson disease (PD). During design, there is often a focus on technical performance, accuracy, and reliability, with less emphasis on the user experience.

**Objective:**

This study explored the user experience of a novel prototype wearable device (wrist- and ankle-worn) to record limb movements (accelerations and angular velocities), and physiological data (eg, photoplethysmography and heart rate information for estimation of blood pressure).

**Methods:**

This qualitative study used internet-based semistructured interviews with people with PD, following wearing prototype devices for 24 hours at home. Interviews were audio-recorded and transcripts analyzed using a hybrid deductive and inductive approach.

**Results:**

Six people with PD, 3 male and 3 female, aged 52-83 years, with mild-to-moderate PD (Hoehn and Yahr scale score ≤3; median MDS-UPDRS [Movement Disorder Society - Unified Parkinson’s Disease Rating Scale] score of 72/199) and cognition within normal limits (6-item Cognitive Impairment Test median score 0), with an average disease duration of 8 years took part in the study. Participants were overall positive toward the device, finding it generally comfortable, light in weight and noninvasive. Five out of 6 participants reported minor problems related to strap adjustability and challenges specific to PD. The prototype was comfortable, but this was a lesser priority than robustness, the device not hindering their usual clothing choices (device size or outward projection, or both), and adjustability for fit, including the need to switch to an elasticated strap. In particular, a discreet design was important, as some individuals may feel self-conscious about wearing visible condition-specific products, due to stigma. The ankle-worn device was perceived as unfamiliar and nondiscreet, with some participants likening it to a prisoner tracking system and dressing specifically to conceal it. The wrist-worn device was considered more user-friendly, especially if designed discreetly and resembling more familiar devices. Five of the 6 participants believed their health care teams should have access to the data, particularly relating to their symptoms, fluctuations, and medication. Three also wished to access these data for self-management. One participant was hesitant regarding the potential benefits of technology to support PD management, preferring to use feedback data personally and relying on their health care team’s usual assessment to guide decisions. Across the group, desirable device technical features included symptom prediction, reminder prompts, and support for medication management. Despite concerns about stigma, most participants were willing to wear PD-specific devices, believing that they could aid better symptom management.

**Conclusions:**

In summary, wearable devices must be discreet, robust, comfortable, and easily applied to promote adherence regardless of technical specifications.

## Introduction

Parkinson disease (PD) is the second most common chronic neurodegenerative disorder, with an increased incidence with aging [1]. PD is characterized by impaired motor function (ie, tremor, bradykinesia, and rigidity) [2], a wide range of nonmotor symptoms (eg, sleep disturbances and fatigue) [3], and by orthostatic hypotension, reflecting autonomic dysfunction and medication side effects [4].

Improving assessment is crucial for monitoring disease progression, adjusting medication doses, enhancing quality of life, and improving access to treatment services for people with PD [5]. Due to the heterogeneity and complexity of PD features, clinical assessment may be challenging and not always consistent as it relies on subjective clinician assessment (eg, using the Movement Disorder Society - Unified Parkinson’s Disease Rating Scale [MDS-UPDRS]) [6] and self-evaluation by patients [7]. Intermittent clinical examinations offer limited understanding of daily symptom fluctuations [8], and formal assessment of PD can often be time-consuming and susceptible to interrater variability [9,10]. Clinical assessments may not always capture patients in their typical condition [11], while self-report can be burdensome and relies on individuals’ recollections, where time elapsed between appointments can make it difficult to recall symptoms [12]. Accessing services also remains challenging due to long waiting lists, infrequent routine clinic visits, and travel for appointments, which can be fatiguing and costly [13].

Wearable devices for daily and remote monitoring of people with PD present opportunities, especially in a health service model that values person-centered and community-based approaches [14]. They can collect continuous, accurate, and objective data, and have been used to detect tremor [15], mobility [16], bradykinesia [17], dyskinesia [18], motor fluctuations [19], gait speed [20], sleep [21], falls [22], and physical activity [23].

Current wearable devices offer PD symptom detection and physiological monitoring (ie, blood pressure [BP]); however, few certified solutions exist for at-home motor symptom monitoring in people with PD. These include Parkinson’s Kinetigraph [24], STAT-ON [25], Kinesia 360 [26], and the Apple watch with StrivePD [27] mobile app. MoveMonitor [28], Actiwatch [29], and ActiGraph [30] products, GT9X Link, and wGT3X-BT are devices that have also been used in validation studies for estimating physical activity, gait parameters, and sleep patterns in people with PD [31-33]. Given the prevalence of orthostatic hypotension, BP monitoring is another important aspect of PD management. The Samsung SM-R850 has been evaluated for BP measurements in people with PD and fulfilled the BP validation criterion of the International Organization for Standardization [34]. To the authors’ knowledge, there are no off-the-shelf systems or studies that incorporate motor symptom and gait speed monitoring with photoplethysmography (PPG) and electrocardiogram (ECG) sensors for simultaneous BP calculation. To address this gap, a prototype wearable device system called the “Wearable Enabled Symptom Assessment Algorithm” (WESAA) has been proposed to collect concurrent inertial, PPG, and ECG raw signal waveforms. In particular, differentiating off-symptoms from hypotension could support clinicians in decision-making to improve quality of life.

Technology research typically focuses on the device’s technical performance, with less evaluation of the user experience [35], which may result in slow adoption of wearable devices into routine clinical care pathways [36]. Mostly, the user experience is based on surveys [37-39], focuses on the clinician perspective [40], or includes mixed patient populations [41]. Rarely do people with PD report their preferences and expectations [42], despite a need to enhance the patient’s engagement in their own care [43]. Wearable device designers who adopt a person- and user-centered design philosophy that takes into consideration users’ involvement in the early stage of design are more likely to enable ongoing user engagement via a device that is suited to the needs of people with PD [35]. Therefore, this study aimed to apply a novel approach by including people with PD in the design process, concurrent with device development, to answer the question: “what are the user experiences of the novel prototype system?” Specific objectives were to:

- assess the usability and acceptability of the WESAA prototype system among people with PD and the practical implications of using it in daily life.
- explore user-interface preferences among people with PD using the WESAA system.
- examine broader attitudes toward wearable technology for PD management and desirable features for future wearable devices.

## Methods

### User-Centered Design

The authors engaged with different stakeholders, including clinicians, people with PD, caregivers, human factors engineering practitioners, and medical device designers to collect perspectives and feedback to ensure a comprehensive perspective and its significance to people with PD. To ensure that the study was grounded in the perspectives of people with PD, there was active involvement of 3 members of the Parkinson Ireland branch, comprising 2 people with PD and a caregiver of a person with PD. This collaboration included: identifying and prioritizing the research questions; reviewing the data collection tools to ensure that these were relevant, sensitive, and user-friendly; determining the duration for which participants wore the device, taking into account factors such as comfort and feasibility; corecruiting participants for the study; and informing effective ways to disseminate the results to the Parkinson community. These collaborators did not participate in the study as participants.

System requirements for the prototype device were developed based on this guidance together with a comprehensive study of the current state-of-the-art and device market. The industrial design of the system was conducted by an external collaborator, Design Partners, Ireland [44], under the direction of the authors. After concept validation, the approach moved to the prototype and testing phase, where a prototype was created to test and refine the proposed solution, and then evaluated with the users in the test phase in order to collect feedback and identify opportunities for improvement.

### Included Devices: The WESAA Prototype System

**Figure 1:**
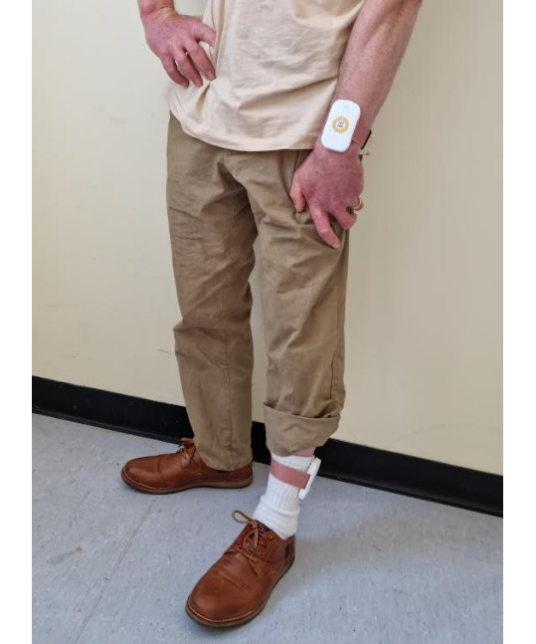
Wearable Enabled Symptom Assessment Algorithm system - wrist and ankle. A usability study of limb-worn wearable devices for monitoring motor function and blood pressure in people with Parkinson disease in Ireland in the period 2021-2023. Photograph of a participant’s lower body with the Wearable Enabled Symptom Assessment Algorithm wearable device system attached to the left wrist and left ankle.

**Figure 2:**
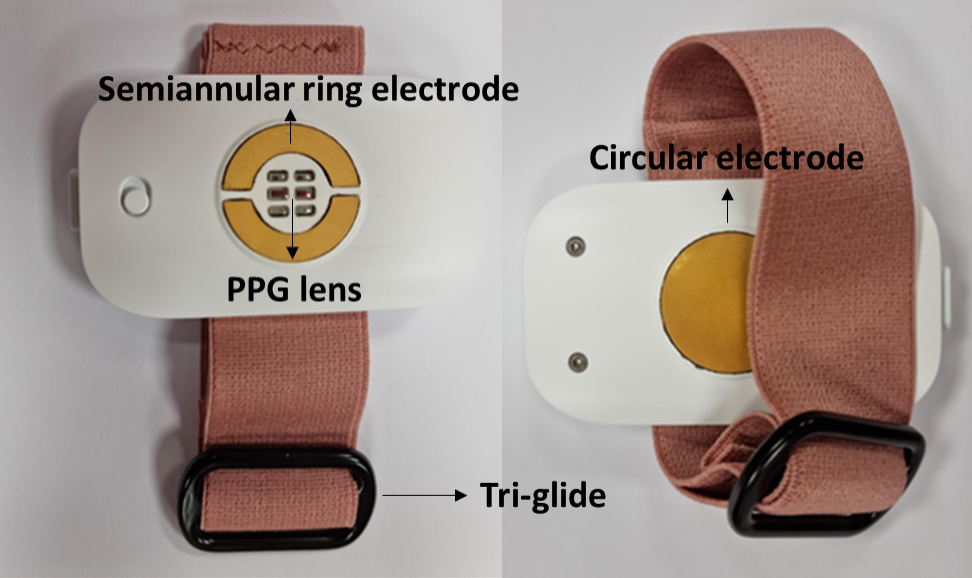
The Wearable Enabled Symptom Assessment Algorithm unit was used in the usability study of limb-worn wearable devices for monitoring motor function and blood pressure in people with Parkinson disease in Ireland in the period 2021-2023. Photograph showing the system from the front and underside. The front view features an orange stretchable band with a tri-glide slide, a white rectangular box containing a semiannular gold ring and a photoplethysmography lens. The underside view reveals the same orange band and white box, now displaying a gold circular electrode. PPG: photoplethysmography.

In the WESAA system, 2 identical units are worn, on the external side of the wrist and ankle of the most affected side (Figure 1). Additional information regarding the design of the WESAA system is available in the publication titled “Design of a Multi-Sensors Wearable System for Continuous Home Monitoring of Individuals with Parkinson” [45]. Each device embeds an inertial monitoring unit to record accelerations and angular velocities as well as PPG and ECG sensors. Each enclosure is 45.3 mm × 76.8 mm × 14 mm, latex-free, weighs approximately 41 g, and is fastened to provide secure contact to reduce artefact. Tri-glide slides, used as buckles, use friction to secure the enclosure in place. The strap is 30 mm wide, latex-free, and weighs approximately 22 g (Figure 2).

Participants wore the system at home, allowing a comprehensive approach where cultural and social activities are considered. Devices were removed during water activities, following guidance on how to remove and put them on. The WESAA system collects, contextualizes, and stores up to 10 days of data from the wrist and ankle using a low-power microcontroller unit within the device. These data, including raw inertial, PPG, and ECG signals, are transferred to a personal computer via a USB cable, where they can be further analyzed, processed, and visualized using sophisticated machine learning and data analytics methods. After a 24-hour period, researchers met with participants to collect the system. The decision to limit the wearing period was influenced by the prototype nature and input from the Parkinson advisors, who suggested that a 24-hour period would be sufficient to gather insights into the devices without being burdensome.

### Recruitment Setting and Participants

Participants were recruited through local branches of Parkinson Ireland, a not-for-profit advocacy and support organization, and through a PD specialist clinic. The researcher (LK, female research assistant trained in qualitative methods, with a social science background) contacted the groups to share study information as appropriate (in-person, word of mouth, and posters in the clinic). Recruitment through Parkinson Ireland involved the circulation of the study details through online support group meetings (COVID-19 era). The researcher attended these meetings and gave an overview of what the study entailed. Recruitment through the PD specialist clinic involved the researcher attending in-person at a Health Service Executive clinical site where patients visit a consultant geriatrician with a special interest in PD. The consultant screened for suitability and initial interest and facilitated introductions between the researcher and patient, where once again an outline of the study was given. Those interested were invited to contact the researcher where the study was further discussed in detail, including details of the nature and duration of the study, and the optional poststudy internet-based interview, the potential risks and benefits involved, and their right to choose not to participate. LK had no prior relationship with study participants, who understood her as the study’s research assistant. Participants satisfying the following criteria were eligible: diagnosis of PD, aged 50 years and older, ambulatory, and capable of fulfilling the study requirements. After wearing the WESAA prototype, a purposive sample of 6 participants who wore the device at home were asked to consider participating in an optional poststudy internet-based interview after they handed back the devices. They took the participant information leaflet and consent form for the interview home and had the period of wearing the device to decide if they wished to take part in the interview. The researcher contacted them the following morning while they were wearing the device to ensure all was well, and this provided an opportunity for further questions, and to arrange the internet-based interview from their home at a time of their convenience, if interested. The signed consent form was brought with them on the day they handed back the devices. Participants were selected using a criterion theoretical sampling strategy to satisfy the following criteria: inclusion of different age categories (50-60 years, 61-70 years, 71-80 years, and >80 years) and the inclusion of men and women. Participants were excluded if they had significant communication deficits. Interviews took place as soon as possible, within a week of wearing the device.

### Qualitative Research Methods

Due to the heterogeneity of PD, it was deemed more suitable to use qualitative research methods for this population, to potentially discover usability barriers that are overlooked by quantitative methods [46]. Internet-based semistructured interviews were chosen as the data collection method to reduce travel burden and costs and offer scheduling flexibility. Microsoft Teams hosted by the university was used, where only individuals with authorized access participated, and a “waiting room” was used, with a controlled experience (eg, attendees cannot change meeting settings or share content). A separate audio recording device was used, where audio files were password-protected in a secure database.

An interview topic guide was designed based on existing literature and the purpose of the study, reviewed by the stakeholders and researchers together in terms of the content, focus, and significance to people with PD. Questions explored the experience of using the wearables, perspectives on the sharing of health-related data, familiarity with wearable devices and technology for PD, perception of wearable devices for PD, and future capabilities of wearable devices for PD (Multimedia Appendix 1). Pilot testing of the interview topic guide was conducted to validate its effectiveness, and subsequently, interviews were conducted using the topic guide to stimulate general discussion. To support the interdisciplinary nature of the research, the interviews involved participation from a member of the research team (LK) and a member of the industrial design collaborators, Design Partners. Interviews lasted between 25 and 35 minutes. All interviews were audio-recorded, transcribed verbatim, and field notes were taken

### Data Analysis

Transcripts were analyzed using NVivo (QSR International Pty Ltd) for common themes using a qualitative thematic approach [47]_._ The researcher (LK) initially used an inductive coding approach within an interpretivist paradigm, where data-derived codes emerged as the researcher navigated through the data. A second researcher (LOM, female researcher with a background in public health) reviewed a sample of transcripts to enhance intercoder reliability and ensure consistency in theme identification. Codes were grouped into provisional subthemes and themes, with key phrases assigned based on content relevance. The alignment of codes with research questions facilitated deductive analysis to ensure comprehensive coverage of study objectives.

Reflexivity was maintained through ongoing reflection on the researcher’s assumptions, perceptions, and interactions with participants. Member checking and peer debriefing enhanced the credibility of the findings. After 4 interviews, a recurring pattern in the responses was observed which indicated that data saturation may have been reached. To ensure rigor, data collection continued until a total of 6 interviews were completed, at which point no new themes or perspectives emerged.

### Ethical Considerations

This study was approved by the Clinical Research Ethics Committee at University College, Cork (ECM 4 (r) 11/10/2020) and consent was obtained prior to participant recruitment. The informed consent process provided information about rights as research participants, study objectives, potential risks and benefits, and confidentiality measures. Written informed consent was obtained from all participants. Participation was voluntary and no compensation was given. Any personal information was anonymized before analysis. While no identifiable facial features are presented, documented written consent was obtained from the participant depicted in the photograph in the publication.

## Results

### Participants

As per Table 1, people with PD, aged 52-83 years, with an average disease duration of 8 years, with mild-to-moderate PD (Hoehn and Yahr scale score ≤3; median MDS-UPDRS score of 72/199) and cognition within normal limits (6-item Cognitive Impairment Test [6-CIT] median score 0), took part in the internet-based interviews, which lasted between 26 and 50 minutes. No invited participant declined.

**Table 1:** Clinical and demographic data of the study participants enrolled in the usability study of limb-worn wearable devices for monitoring PD motor function and blood pressure in Ireland in the period 2021-2023 (N=6).

Clinical and demographic data		
Participants, n (%)		6 (100)
**Sex, n (%)**		
	Male	3 (50)
	Female	3 (50)
Age (years), median (IQR; range)		66 (12.5; 52-83)
BMI, median (IQR; range)		24.8 (3.14; 23.94-33.79)
Hoehn and Yahr stage, median (IQR; range)		2 (0; 2-3)
Years since diagnosis, median (IQR; range)		7 (4.5; 3-17)
MDS-UPDRS^a^ (total score), median (IQR; range)		72 (18.5; 38-78)
MDS-UPDRS III (motor examination score), median (IQR; range)		36.5 (23.5; 7-45)
6-CIT^b^, median (IQR; range)		0 (0; 0-4)

^a^MDS-UPDRS: Movement Disorder Society - Unified Parkinson’s Disease Rating Scale.

^b^6-CIT: 6-Item Cognitive Impairment Test.

Five themes were identified in the analysis: (1) Life with Parkinson Disease, (2) Usability and Acceptability Factors of the WESAA System, (3) User interface and Data Access, (4) Attitudes toward Wearable Technology for PD, and (5) Additional Desirable Features of a Wearable Device. These are summarized in Table 2. To ensure anonymity while reporting, participants are labeled as P1, P2, P3, P4, P5, and P6.

**Table 2:** Summary of Thematic Topic Areas. A usability study of limb-worn wearable devices for monitoring motor function and blood pressure in people with PD in Ireland in the period 2021-2023.

Theme	Subtheme
Life with Parkinson Disease	PD^a^ is an impactful condition, with symptom variability and unpredictability, requiring adjustments of routine and pharmaceutical reliance. Management techniques include adjusting medication and physical activity. A team-based approach is essential. Social challenges such as social isolation and stigma contribute to loneliness and employment difficulties.
Usability and Acceptability Factors of the WESAA^b^ System	Participants had a positive attitude toward the testing. The device was comfortable and noninvasive. Usability challenges included device size (with clothing), strap issues, and water resistance; the system was not discreet.
User Interface and Data Access	Desire for feedback on system functioning and data upload status. Suggestions for a user-friendly interface to enhance usability and provide real-time feedback. Preferences varied regarding sharing data with health care teams, highlighting individual preferences; emphasis on health care professionals (not patients) making clinical decisions.
Attitudes toward Wearable Technology for PD	Positive attitude toward wearable technology in general. Perceived benefits such as accurate symptom recording and improved communication with health care teams. Wearables were seen as a source of motivation for physical activity and a tool to enhance self-monitoring.
Additional Desirable Features of a Wearable Device	Wearable technology should be personalized to individual needs. Recommendations include personalized tracking of health metrics such as blood pressure, sleep habits, and medication levels. Value in reminder prompts for medication, exercise, and symptom management. Continuous monitoring is recommended for fine-tuning medication. Suggestions to include additional features such as gait and balance tests, step counters, voice therapy applications, and cognitive training tools.

^a^PD: Parkinson disease.

^b^WESAA: Wearable Enabled Symptom Assessment Algorithm.

### Topic Area 1: Life With Parkinson Disease

The first theme, “Life with Parkinson Disease,” provides contextual background information that enriches the understanding of participants’ experiences wearing the devices. This theme offers insights into the daily challenges, routines, and activities people with PD navigate, which can impact their perceptions of and interactions with wearable devices. Understanding the broader context of participants’ lives is crucial for interpreting their responses and identifying potential barriers and facilitators to device use in real-world settings.

Participants expressed the profound impact of a PD diagnosis, requiring ongoing treatment and adjustments to daily routines. Common challenges included symptom fluctuations and unpredictability, with changes in both motor and nonmotor symptoms throughout the day, which might impact the ability to use a device.

You might get out of bed in the morning and say forget it, I'm not able to do what I want to do today. And then you might get out of bed in the morning and you might be absolutely fine and get much more done than you think; so it's you take each day as it comes.

P4

Many discussed strategies of adjusting medication timing and dosage, trialing new medications or therapies, and exercise and physical therapy in attempts to improve functioning*.* A device needs to support and not impede these goals.

Some felt that PD can be:

…very isolating when you’re by yourself.

P3

Tremors, stiffness, and difficulty with movement make it challenging to perform everyday tasks, contributing to feelings of loneliness, isolation, and social stigma. PD symptoms together with social discomfort can create barriers to social interaction, leading to increased feelings of isolation in people’s work and social lives:

The concern would be that people know I have Parkinson's, then they might not want me to do their work, because they might trust me to do it, even though I'm perfectly capable of doing it.

P2

Thus, a device needs to be discreet.

### Topic Area 2: Usability and Acceptability Factors of the WESAA System

All participants expressed a positive attitude toward testing the prototype system for research purposes, eagerly volunteering to take part in the study:

I'm optimistic that this advancement today is going to bear fruit in time.

P1

While wearing the system, participants engaged in varied activities—some active, such as cycling and socializing, and others at home reading or watching TV.

The system was considered acceptable, generally comfortable, light in weight, and noninvasive. While generally satisfactory, significant practical implications were identified in terms of usability. The size of the current devices was found to be “bulky” especially in relation to their outward projection:

…the only thing that would strike me about it…is certainly is big. If you could reduce that in size again, I think it would help.

P5

Some felt that sleep was disrupted; one person experienced intermittent sleep with 1 or 2 awakenings, while another was aware of the devices initially but slept through without interruptions.

Devices were fastened using a latex-free stretchy band, and participants had different experiences with adjusting, putting on, and taking off. One person had no issues, but others reported minor problems related to strap adjustability and challenges specific to PD. In some instances, the strap became loose over time and it was challenging to keep the devices in place, affecting device stability. In contrast, some experienced a tightening of the strap, which made it difficult to reduce the tension of the strap when adjusting or removing.

The plastic adjuster slider used to adjust the tightness of the strap posed a challenge for some due to PD-related barriers such as tremors, slowness, and fluctuations, where

manipulating the strap... it was a little bit difficult.

P6

Future designs should incorporate a strap that is secure, less inclined to loosen, easier to adjust, and manipulate. Suggestions included features familiar to people with PD, such as Velcro or a watch-style strap as these could be used with one hand, without the assistance of others.

The prototype was not completely sealed and was not water-resistant, which impacted robustness:

I think the bottom line is that the devices can take a lot of punishment, but they can't take too much.

P1

Some found it challenging to be careful with movement for fear of damaging which in turn would affect the technical performance, “I was conscious all the time that I had it” (P3); thus, some participants altered their usual behaviors while wearing.

While generally comfortable, the challenge of making it work with clothing and adjusting it for a proper fit outweighed the comfort factor. The devices’ size and outward projection meant, “*you'd have real problems getting a sleeve on over the [device]... or a tight pair of jeans if you wear on the leg*” (P4).

Moreover, devices were found to be nondiscreet, with the potential to draw attention to the wearer where “it was very obvious, because it is quite clunky and unusual looking” (P2).

There was a perceived social and emotional impact from this as not all people with PD openly communicated their condition, and there were concerns that a device could disclose a person’s health status:

I'd be very conscious of having it. Particularly the arm one visible, because people are going to say, ‘Oh, that's a strange looking watch,’ and then you're trying to explain.

P4

Some had a negative perception of the ankle-worn device which was regarded as unfamiliar because “very few people wear something on their leg” (P6).

One person dressed in such a way as to conceal and another likened it to a prisoner tracking system saying:

I was afraid I'd be labelled some kind a sex offender or some kind of prisoner.

P2

The wrist-worn device was considered more user-friendly for long-term use, especially if it was made smaller, more discreet, and resembled more universally familiar devices.

### Topic Area 3: User Interface and Data Access

There was a desire to receive information on the system’s functioning status, namely whether it was recording or positioned correctly as participants never got feedback as to whether data successfully uploaded or not*,* and so a system that needed special attention had the potential to be troublesome:

There was a slight bit of anxiety on my part as to whether I'd screwed up the whole thing by nudging it off something.

P2

In addition, some participants were interested in accessing the health-related data collected, suggesting the inclusion of a user interface would enhance satisfaction.

Most believed that health care teams should have access to the collected data, but 1 participant expressed hesitation regarding the potential benefits of technology in managing their PD, preferring to keep information solely to themselves, believing that technology and health care should be kept as “two separate systems” and they “would go along with the (health) team as they are telling them” (P3).

Three participants wished for both themselves and their health care teams to receive the data, and were interested in sharing data relating to their symptoms, fluctuations, and medication as “they’re capable bits of equipment that could collect information for my doctor’ (P1).

Providing clinicians with access to data would aid in gaining a better understanding of their condition, with different methods suggested for achieving this goal. One saw information being given first to health care teams and then fed back to the people with PD:

…because then the consultant can say, well, we can see this is happening or that's happening. And then you understand where you're at more yourself.

P4

Another felt that health care teams would not always have the capacity to review data daily or weekly and therefore would like access themselves first, and then health care teams thereafter could view at intervals. Although having access to their own data was important, individuals still wanted input from their health care teams, as “there's only so much I can do with the information, but they could analyse it better” (P6).

Technology was seen as an additional tool for receiving adequate health care, but it should not replace the essential role of health care teams.

Two participants preferred to have their information relayed solely to their health care team. They explained that they did not want to be constantly reminded of their PD:

I'm a bit like the ostrich. I have this illness, I stick my head in the sand and I change the philosophy. Unless I need to know, I don't want to know.

P5

### Topic 4: Attitudes Toward Wearable Technology for PD

Participants recognized the integration of wearable devices in the management of PD, as suitable technology could benefit their daily lives. Objective recording of disease symptoms was cited as one of the benefits, and predicting symptoms, particularly real-time ON and OFF states, could enhance medication management. Managing the condition can become overwhelming and that:

…it would be lovely to have something recording and because we've so much to think of, exercises, tablets, and keeping up your spirits. Each and every day, it would be great to have something working in the background to record what's actually happening.

P6

The system encouraged some to be more active where 1 participant mentioned that:

I suppose it could keep you active and keep you informed of what you're doing, and what improvements are there.

P3

As time with health care professionals was perceived to be limited, wearable devices were seen as a solution to reinforce communication with data and provide confidence when attending appointments. Recorded health parameters could be communicated to health care teams allowing them “to be more precise in their assessment of what is wrong and what can be done to lessen the impact of the illness” (P5), reducing the burden of people with PD self-recall. If health care teams had better access to the health data they would have a clearer understanding of the current and future projected state of people with PD:

…so I would be hopeful that it would bridge the unknown thing, where I'm at with my condition and where the clinician maybe doesn't quite have the full picture.

P1

There was potential to help regain confidence by providing clarity about their condition, which they can then act upon, as reflected here:

…but then you have confirmation that it is, you actually will get it treated.

P6

Although aspects of the current prototype may require refinement, participants were still willing to wear it if it meant that their health condition could be improved through its use:

I would be happy for the nuisance of it if it gave the consultant that type of information to help them determine how best you can treat my illness.

P5

### Topic 5: Additional Desirable Features of a Wearable Device

Recommendations were made that wearable devices might need to be customized to the particular preferences of people with PD. A one-size-fits-all approach might not work due to the condition’s diversity since there might be important variations between those with early-stage disease and those with more advanced stages. An individualized system that can monitor specific health parameters, provide personalized feedback, and assist with the management of medical conditions would be highly valuable in promoting patient engagement and satisfaction.

Features to incorporate into future wearable devices included enhanced monitoring and therapy provision. One monitoring suggestion was the BP monitoring feature of the WESAA system. Reminder prompts could be advantageous, not only for medication intake, exercise, and movement but also for anticipating symptom fluctuations. The potential usefulness of the ability to fine-tune medication, for example, the possibility of including a dopamine level monitor, comparable to the continuous glucose monitoring used in diabetes, was proposed:

Is there some possibility that a device like that would help monitor your levels of dopamine?

P2

Monitoring sleep was another potentially useful feature, as sleep disturbances can be common in people with PD. Incorporating a gait and balance test application was recommended, especially for those prone to falling or who have balance issues. A step counter was proposed for those interested in physical activity levels, as it was felt that regular physical activity can help improve balance, mobility, mood, and overall physical well-being in PD. People with PD may experience challenges with speech, such as reduced volume and impaired articulation, and integrating voice therapy and brain training applications could provide beneficial therapy, offering a platform for voice maintenance and cognitive development.

## Discussion

### Principal Findings

The success of wearable devices is not only determined by their technological capabilities but also by their usability and wearability components. We examined the experience of people with PD in using the newly developed prototype system known as the WESAA. All participants reported a positive perception of the system, aligning with previous research where participants showed willingness and positivity to wearing complex wearable systems [48], and that any minor inconvenience in wearing the device was outweighed by potential value [41].

While the system was comfortable to wear, the size and outward projection of the individual devices were too large. Consistent with previous research [35,48], a device that was small, easy to fasten, noninvasive, waterproof, and durable was preferred.

Device fastenings used a latex-free stretchy band, and in some instances, the strap became loose and affected device stability. Similar to AlMahadin et al [12], Velcro straps were the most desired fastening mechanism. Ensuring a device does not have a negative impact on the user’s daily routines is crucial [12]. When a user’s routine is disturbed, particularly among older users, the device faces challenges in seamlessly becoming an essential part of the user’s life and potentially resulting in a loss of its perceived value [49]. Incorporating wearable devices into the existing behavior of people with PD should be seamless, without requiring the learning of new practices or routines.

There is a recognized stigma associated with PD [50], highlighting the importance of discretion in future design. Our study revealed concerns about the potential to draw attention to the wearer, leading to feelings of embarrassment and self-consciousness, particularly if it revealed their condition. This could affect willingness to continue monitoring beyond the initial 24-hour period. Prioritizing ease of use and aesthetic appeal is important, as the latter appears to be closely linked to stigma concerns. Interestingly, previous research by our team [43], where people with PD considered the hypothetical wearing of various wearable devices, found that while the size and weight of the devices were crucial, aesthetics were deemed less significant (ie, “function over form”). Moreover, AlMahadin et al’s study [12] showed that participants had no concerns about device visibility and actually preferred to be identified as a person with PD, signaling that they may require assistance. Our research reveals a contrasting preference for devices that were discreet, which perhaps reflects the reality of wearing a system for 24 hours in daily life. Previous research [37,41] demonstrated that people with PD can be uncomfortable with an ankle-worn device, a sentiment expressed by our participants, considering it unfamiliar, and even comparing it to a prisoner tracking system. Thus, the incorporation of an ankle device into a PD monitoring system would need consideration by designers if they want to ensure acceptance and compliance.

Responses revealed a consensus on the benefits of sharing health-related data with health care teams; however, a subset of individuals also expressed a desire for personal ownership of their information and the ability to access it themselves, while others preferred not to be constantly reminded of their PD condition. A combination of health care and self-care in managing any chronic condition has been highlighted [51], and for wearable systems in PD to achieve their maximum effectiveness, people with PD who wish should have regular access to their personalized health-related data and ideally, this access should be in real time. Hence, designers face a challenge in developing systems that can meet the diverse preferences regarding access to health care information on a wearable device. An additional task is to meet the stated diverse needs for additional features in a wearable system, extending from enhanced monitoring of several parameters to therapeutic applications, all in a smaller device.

Reliable, continuous, and objective recording of individual health parameters that can be shared with health care teams has been recognized by people with PD [12]. The majority expressed support for the exchange of information between people with PD and their health care team, via accurate and objective recording of disease symptoms. It was felt there was limited access to clinical consultations for people with PD; however, WESAA offers an opportunity to attend appointments confidently by reinforcing communication with reliable objective data. These data can be accessed by clinicians to support people with PD to accurately recall symptoms, improving the quality of communication during consultations and reducing the burden of self-recall.

A wearable system tailored to people with PD carries the hope of empowering patients by minimizing the disruption caused by “health care inconveniences” in their day-to-day lives and potentially reducing the need to spend time recording symptoms and travel for appointments [52]. Furthermore, gathering data continuously offers a more comprehensive dataset compared to the snapshot readings obtained during visits to health care facilities [53]. Those with greater levels of health information are equipped to contribute more valuable inputs concerning their condition, therefore encouraging collaborative decision-making and facilitating adherence to agreed-upon plans [54].

### Strength and Limitations

The research explored the practical application of wearable devices within a domestic 24-hour setting, as opposed to hypothetical perspectives or limited laboratory or clinical testing. It is important to acknowledge the limitations imposed by the small sample size and the recruitment from a PD support group, which may not represent the broader population of people with PD. While data saturation was reached, PD is a heterogeneous disease, and it is possible that these findings may have been subject to refinement if more perspectives were captured across different disease stages and phenotypes, and across more diverse age and cultural profiles. This study was in its infancy and research questions were still emerging. Future research should aim for a larger and more diverse sample through broader sampling, where new insights might emerge.

Acknowledging the limitations of the 24-hour wearing period is practical, largely in terms of capturing long-term or nuanced user experiences. As devices evolve, future studies may consider longitudinal study designs to gain deeper insights into users’ experiences over time. Nonetheless, the chosen testing period was deemed appropriate for the goals of the study and the stage of prototype development.

As the interviews conducted were internet-based interviews—where there was limited visual interaction and nonverbal communication may have been missed. Participants had no technological engagement with the system; therefore, any bias regarding specific technical features was unlikely. Going forward, it might be useful to involve participants with varying levels of familiarity with technology to explore potential differences between those who are tech-savvy versus those less familiar with technology.

While no formal clinical safety testing (eg, dermatological assessment for skin irritation or pressure marks) was conducted, no user reported itch, redness, rash or other irritation. To ensure real-world adherence and safety, all future design developments should also include strap safety testing.

### Conclusion

The study involved conducting research and progressively testing designs with people with PD. A deeper understanding of the requirements of people with PD, the problem domain, and the technological options available was obtained. This helps facilitate the future development of more sophisticated and targeted prototypes for people with PD. The study demonstrates that designers must take into consideration concerns of size, discretion, and strap design and be willing to include additional user interface elements in accordance with the user’s preferences.

## Appendix

Multimedia Appendix 1 Interview guide.

## Abbreviations

6-CIT: 6-item Cognitive Impairment Test
ECG: electrocardiogram
MDS-UPDRS: Movement Disorder Society - Unified Parkinson’s Disease Rating Scale
PD: Parkinson disease
PPG: photoplethysmography
WESAA: Wearable Enabled Symptom Assessment Algorithm

## Data Availability

The datasets generated or analyzed during this study are not publicly available due to information that could compromise the privacy of research participants, but are available from the corresponding author on reasonable request.
