# Supplementary material for: User Experiences of Limb-Worn Wearable Devices for Monitoring Parkinson Disease Motor Function and Blood Pressure: Usability Study
Source: JMIR Form Res. 2025 Nov 19;9:e73423. doi: 10.2196/73423 (PMC12676217; doi:10.2196/73423)
Supplement: Multimedia Appendix 1 [file formative_v9i1e73423_app1.docx]

Appendix 1: Interview Guide

Thank you for signing the consent form to take part in this interview. My name is < > and I will be moderating today’s session.

Do you have any questions about taking part in today’s interview?

The main purpose of this interview is to get feedback from you on the devices you have been wearing on your ankle and wrist as part of the study. This might help us to make changes that make them better, if they needed. So please be as honest as you like; we’re evaluating the product not the person using them.

Can I confirm that I have your permission to record this interview so that I don’t have to take detailed notes as we talk? I’ll ask you to repeat that once I turn on the recording. *Recording begins.*

Please do ask me to repeat anything you didn’t hear or understand. We can take a break whenever you like.

|  |  |  |
| --- | --- | --- |
| 1 | Could you describe to me a typical day wearing the devices at home? What did you do? |  |
| 2 | How was your experience wearing the devices doing these?  (Prompt: Positive or Negative?  Why did you say this? |  |
| 3 | Are the devices intuitive/ easy to understand? (examples given) |  |
| 4 | Did you experience any difficulties wearing the device? (*eg getting caught in clothes or worried about damaging them or getting them wet?).*  What was the cause of this?  Did this seem to relate to the device itself*?*  Did your Parkinson’s ever affect your ability to engage with the product?) |  |
| 5 (I) | 1. On a scale of 1-5 (1 being very easy, 5 being very difficult) how hard was it to perform these tasks? (They have received the scale and have it as a prompt with them when answering) 2. Use the device (Taking your blood pressure measurement)   *(For each answer, probe whenever a score of 4 or 5 given – prompt: issue with the device and any PD-specific issues?)*  *Is there anything you would change about the devices? Why? Is this “nice to have” or essential?* |  |
| 5 (II) | Wear it during the day and evening  *(For each answer, probe whenever a score of 4 or 5 given – prompt: issue with the device and any PD-specific issues?)*  *Is there anything you would change about the devices? Why? Is this “nice to have” or essential?* |  |
| 5 (III) | Wear it while sleeping  *(For each answer, probe whenever a score of 4 or 5 given – prompt: issue with the device and any PD-specific issues?)*  *Is there anything you would change about the devices? Why? Is this “nice to have” or essential?* |  |
| 5 (IV) | Put the device on  *(For each answer, probe whenever a score of 4 or 5 given – prompt: issue with the device and any PD-specific issues?)*  *Is there anything you would change about the devices? Why? Is this “nice to have” or essential?* |  |
| 5 (V) | Remove the device  *(For each answer, probe whenever a score of 4 or 5 given – prompt: issue with the device and any PD-specific issues?)*  *Is there anything you would change about the devices? Why? Is this “nice to have” or essential?* |  |
| 5 (VI) | Turn the device on/off  *(For each answer, probe whenever a score of 4 or 5 given – prompt: issue with the device and any PD-specific issues?)*  *Is there anything you would change about the devices? Why? Is this “nice to have” or essential?* |  |
| 6. | Is there anything you would change about the devices? Why?  Is this “nice to have” or essential? |  |
|  |  |  |
| 7 | How would you feel about wearing devices such as this in everyday life?  Why do you say this? |  |
| 8 (I) | Have you worn devices like this before? |  |
| 8 (II) | Do you use anything else to help manage your health or medications in your everyday life? |  |
| 9 | What is your overall perception of these devices?  (Prompt to consider both the wrist and ankle device) |  |
| 10 (I) | How do you feel in general about wearing a device that that monitors your Parkinson’s?  Please elaborate |  |
| 10 (II) | Would your priority be that it informs your clinical team, or that it helps you to better understand your condition?  Please elaborate |  |
| 10 (III) | What about it helping researchers to better understand Parkinson’s?  Please elaborate |  |
| 11 | Do you want to be more involved in managing your Parkinson’s or are you happy for your treatment team to make the decisions?  Please elaborate |  |
| 12 | If there was one thing that you could know on a daily basis that would improve your ability to manage your condition, what would it be?  Nice or essential |  |
| 13 | Is there anything we haven’t discussed today that you would like to discuss? |  |
| 14 | Do you have any questions? |  |
